# Targeting the *Plasmodium falciparum* UCHL3 ubiquitin hydrolase using chemically constrained peptides

**DOI:** 10.1073/pnas.2322923121

**Published:** 2024-05-13

**Authors:** Harry R. King, Mark Bycroft, Thanh-Binh Nguyen, Geoff Kelly, Alexander A. Vinogradov, Pamela J. E. Rowling, Katherine Stott, David B. Ascher, Hiroaki Suga, Laura S. Itzhaki, Katerina Artavanis-Tsakonas

**Affiliations:** ^a^Department of Pathology, University of Cambridge, Cambridge CB2 1QP, United Kingdom; ^b^Department of Pharmacology, University of Cambridge, Cambridge CB2 1PD, United Kingdom; ^c^School of Chemistry and Molecular Biosciences, University of Queensland, Brisbane QLD 4067, Australia; ^d^NMR Centre, Francis Crick Institute, London NW1 1AT, United Kingdom; ^e^Department of Chemistry, Graduate School of Science, University of Tokyo, Tokyo 113-0033, Japan; ^f^Department of Biochemistry, University of Cambridge, Cambridge CB2 1GA, United Kingdom

**Keywords:** *Plasmodium*, UCHL3, DUB, peptide, ubiquitin

## Abstract

The ubiquitin–proteasome system is essential to all eukaryotes and has been shown to be critical to parasite survival as well, including *Plasmodium falciparum*, the causative agent of the deadliest form of malarial disease. Despite the central role of the ubiquitin–proteasome pathway to parasite viability across its entire life-cycle, specific inhibitors targeting the individual enzymes mediating ubiquitin attachment and removal do not currently exist. The ability to disrupt *P. falciparum* growth at multiple developmental stages is particularly attractive as this could potentially prevent both disease pathology, caused by asexually dividing parasites, as well as transmission which is mediated by sexually differentiated parasites. The deubiquitinating enzyme PfUCHL3 is an essential protein, transcribed across both human and mosquito developmental stages. PfUCHL3 is considered hard to drug by conventional methods given the high level of homology of its active site to human UCHL3 as well as to other UCH domain enzymes. Here, we apply the RaPID mRNA display technology and identify constrained peptides capable of binding to PfUCHL3 with nanomolar affinities. The two lead peptides were found to selectively inhibit the deubiquitinase activity of PfUCHL3 versus HsUCHL3. NMR spectroscopy revealed that the peptides do not act by binding to the active site but instead block binding of the ubiquitin substrate. We demonstrate that this approach can be used to target essential protein–protein interactions within the *Plasmodium* ubiquitin pathway, enabling the application of chemically constrained peptides as a novel class of antimalarial therapeutics.

## Significance

Despite the pivotal importance of the ubiquitin–proteasome pathway to the survival of *Plasmodium*, the parasite causing malaria, there is a notable absence of specific inhibitors targeting enzymes responsible for ubiquitin attachment and removal. The deubiquitinating enzyme PfUCHL3, essential across human and mosquito developmental stages, has proven challenging to target conventionally due to homology with human UCHL3 and other UCH domain enzymes. This study leverages RaPID mRNA display technology to identify selective, chemically constrained peptides with nanomolar affinities for PfUCHL3 that obstruct its interaction with ubiquitin. This approach demonstrates the feasibility of targeting essential protein–protein interactions within the *Plasmodium* ubiquitin pathway, introducing chemically constrained peptides as a novel class of antimalarial therapeutics holding promise for preventing both disease pathology and transmission.

Drug resistance to frontline malaria therapeutics is rapidly increasing, threatening our ability to limit the spread of the parasite and the debilitating disease it causes (1–5). As such, novel approaches to control parasite growth and transmission are urgently needed. The ubiquitin–proteasome system is essential to all eukaryotes and has been shown to be critical to *Plasmodium* survival across its entire lifecycle. Despite this central role to viability, specific inhibitors targeting the individual enzymes that mediate ubiquitin attachment (E1, E2, and E3) and removal [deubiquitinases (DUBs)] do not currently exist. The ability to disrupt *Plasmodium falciparum* growth at multiple developmental stages is particularly attractive as this could potentially prevent both disease pathology, caused by asexually dividing parasites, as well as transmission, which is mediated by sexually differentiated parasites (6–8). The deubiquitinating enzyme PfUCHL3 is one such example. It is an essential protein, transcribed across both human and mosquito developmental stages. We have previously characterized this enzyme as a dual DUB/deNeddylase and determined its crystal structure in both Ub-bound and unbound states (9–12). PfUCHL3 is considered to be an “undruggable” target given the high level of homology of its active site to human UCHL3 as well as to other UCH domain enzymes. As such, small-molecule approaches to inhibit its activity would likely be cross-reactive. Moreover, the rest of the protein structure lacks defined pockets where small molecules could bind.

Small molecules and peptides aimed at disrupting protein–protein interactions have shown significant promise as therapeutics in both chronic and communicable diseases (13–17). An advantage of peptide-based drugs is the larger surface of interaction with their therapeutic target as compared to small molecules, which in turn leads to very high-affinity and specific binding and, thus, fewer off-target effects. Moreover, small molecules commonly require deep binding pockets in order to function as specific inhibitors (18). Peptides can target what is known as “undruggable” proteins, those that lack a suitable binding pocket but have much larger and flatter surfaces where protein or nucleic acid partners bind (19–22). Short linear peptides (<50 amino acids) containing only canonical amino acids make poor therapeutic compounds primarily due to their low biostability and bioavailability. Peptide macrocyclization and inclusion of noncanonical amino acids have been shown to substantially increase proteolytic stability, cell permeability, and binding affinity, while also expanding the available chemical diversity (21, 23, 24). Efforts to develop and deliver these types of drugs in the context of malaria infection are ongoing. There are several examples of peptides that have been found to inhibit *P. falciparum* proteins, such as the 13-residue β-hairpin peptide CFTTRMSPPQQIC that inhibits the interaction between the parasite’s apical membrane antigen 1 (AMA1) and the host cell’s rhoptry neck protein (RON) thus blocking the parasite’s ability to invade new erythrocytes (25). Cyclomarin A is a cyclic heptamer found to inhibit *P. falciparum* diadenosine triphosphate hydrolase which inhibits parasite growth (26). Mahafacyclin B is a naturally occurring, cyclic heptamer identified from the latex of *Jatropha mahafalensis* and found to affect parasite viability during the asexual stage (27). A more recent example is a modified tetrapeptide, “3j,” containing a boronic acid warhead in place of the C-terminal carboxyl group which binds to the *P. falciparum* enzyme SUB1. The warhead attacks the active site Ser606 of PfSUB1 and covalently inactivates the enzyme which renders *Plasmodium* asexual-stage parasites unable to undergo egress, effectively blocking reinfection to other red blood cells (28).

Here, we apply the random nonstandard peptides integrated discovery (RaPID) display technology to identify constrained peptides capable of binding to PfUCHL3 with nanomolar affinities (16, 29–32). The two lead peptides were found to selectively inhibit the deubiquitinase activity of PfUCHL3 versus that of HsUCHL3. NMR spectroscopy revealed that the peptides do not act by binding to the active site but instead block the interaction of PfUCHL3 with the ubiquitin substrate. We demonstrate that these approaches can be used to target essential protein–protein interactions within the *Plasmodium* ubiquitin pathway, enabling the use of chemically constrained peptides as a novel class of antimalarial therapeutics.

## Results

### mRNA Display Screening Identifies Peptides That Specifically Bind to PfUCHL3.

**Fig. 1.**
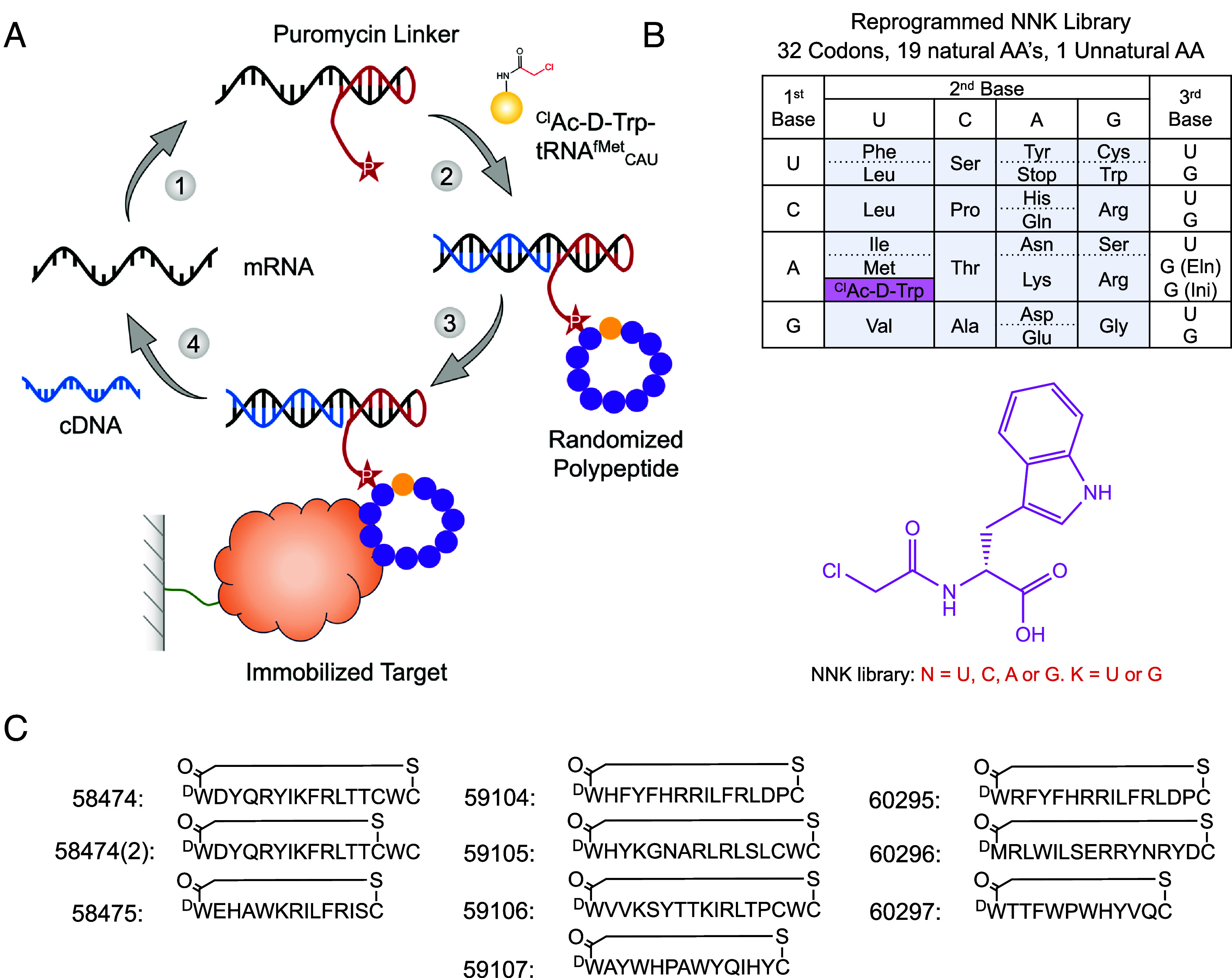
RaPID methodology peptide selection against PfUCHL3. (*A*) In the RaPID technology, an mRNA library is ligated to a puromycin linker (1), translated and reverse transcribed (2), selected against an immobilized protein of interest (3), and the cDNA is recovered to sequence and regenerate a new mRNA library for a subsequent round of selection (4). (*B*) Summary of the NNK genetic code used in the randomized region of the peptide library. The pink box shows the unnatural amino acid which was genetically reprogrammed to replace the initiator methionine [AUG (Ini)] using flexizyme technology, the elongator methionine [AUG(Eln)] was left unprogrammed. (*C*) The top 10 most enriched peptide motifs from selections against PfUCHL3.

In order to maximize the size of the peptide library, we used the RaPID platform, an in vitro mRNA display selection technique that circumvents the need for in vivo transformation steps and allows for the incorporation of nonproteinogenic amino acids to increase chemical diversity. During in vitro translation, each peptide is covalently linked to its cognate mRNA via a puromycin linker. After ribosomal release, the peptide–mRNA is reverse-transcribed to generate a peptide–mRNA–DNA complex. When scaled up and performed in a combinatorial format, libraries in the order of 10^14^ unique peptides can be generated and screened for biological activities of interest (Fig. 1). By using “flexizymes,” small (45 to 46 nucleotides) artificial aaRS-like ribozymes, tRNA substrates can be aminoacylated with unnatural amino acids (UAAs) of choice (29). To conduct genetic code reprogramming in the RaPID system, the natural tRNAs and amino acids for the codon being reprogrammed are omitted from the translation mixture and replaced with engineered tRNAs for that codon which have the desired UAA preloaded onto them by the appropriate flexizyme. In our case, the mRNA library is designed to reassign N-formyl-methionine as N-chloroacetyl-D-tryptophan as the translation initiator. The peptides also feature a carboxy-terminal cysteine such that after translation, they spontaneously cyclize forming a nonreducible thioether bridge (33). Macrocyclic peptides are more conformationally constrained compared to their linear counterparts and therefore often possess higher substrate specificity and affinity. The nonreducible, D-stereochemistry cyclization used in the RaPID system leads to the peptides with increased proteolytic stabilities. During RaPID, peptides undergo repeated rounds of selection, whereby they are screened against the protein of interest, in our case PfUCHL3, which had been immobilized onto streptavidin-coated Dynabeads via a biotin tag (*SI Appendix*, Fig. S1). PfUCHL3 was cloned with an N-terminal AviTag™, GLNDIFEAQKIEWHE, between the protein and a 6xHIS tag. Purified BirA enzyme recognizes the AviTag™ sequence and was used to catalyze the monovalent biotinylation of PfUCHL3 at a location distal to the protein’s surface.

Following seven rounds of selection against PfUCHL3, the peptide library had reached convergence, and 10 peptides from unique peptide families that displayed high sequence enrichment as measured by the next-generation sequencing were selected for synthesis (Fig. 1*C*). The peptides ranged from 12 to 17 amino acids in length and were synthesized commercially in their cyclized state. Since peptides 59105, 59106, and 58474 contained an additional internal cysteine residue, there was potential for two cyclization isomers. Peptides 59105 and 59106 appeared to purify solely as a single species, suggesting that one isomer is the more favorable (likely the peptide cyclizing to the cysteine nearest the N terminus) or that the two different cyclization states coelute and can not easily be separated. However, for peptide 58474, the HPLC trace revealed that both cyclization states were present and could be purified as two different peptides for downstream analysis, peptide 58474 (the major product) and peptide 58474(2) (the minor product).

### Macrocyclic Peptides Bind to PfUCHL3 with High Affinity.

The affinities and kinetics of binding of PfUCHL3 to the peptides were analyzed by biolayer interferometry (BLI). Most of the peptides showed binding, albeit with a wide range of *K*_d_ values from low nM to mid-μM (Table 1). Some peptides (59105, 59106, 60295, and 60297) displayed clear biphasic binding profiles that were fit to a heterogeneous binding model, while others (59484, 59104, and 60296) could be fit to a simpler 1:1 model. Inspection of the tightest *K*_d_ for each peptide indicated that 60296 and 60297 have the highest affinity (9 nM and 2 nM, respectively), followed by 58484 and 59105 (high nM), and the rest are in the μM range.

**Table 1.** Thermodynamic and kinetic parameters underpinning the selection of two lead peptides

Peptide	*K*_d_	*k*_on_ (×10^4^ M s^−1^)	*k*_off_ (×10^−4^ s^−1^)	Δ*T*_m_ (°C) PfUCHL3 (+6 μM peptide)
58474	101 ± 7 nM	1.71 ± 0.09	17.4 ± 0.9	+1.2 ± 0.14
58474(2)	Low	Low	Low	+1.3 ± 0.08
58475	Low	Low	Low	+0.5 ± 0.15
59104	170 ± 5 nM	1.81 ± 0.05	30.7 ± 0.4	−0.4 ± 0.04
59105	~9 μM^*^			+0.1 ± 0.06
59106	~28 μM^*^			−0.4 ± 0.03
59107	NSB	NSB	NSB	+0.6 ± 0.13
60295	~17 μM^*^			−0.2 ± 0.10
60296	9.32 ± 0.28 nM	56.9 ± 1.6	53.0 ± 0.6	+3.1 ± 0.06
60297	~2 nM^*^			+5.1 ± 0.21

^*^Complex profiles required fitting to a heterogeneous model; the tighter of the two *K*_d_ values is shown. NSB = nonspecific binding to the reference sensor, precluding analysis. Low = low binding signal, precluding analysis.

**Fig. 2.**
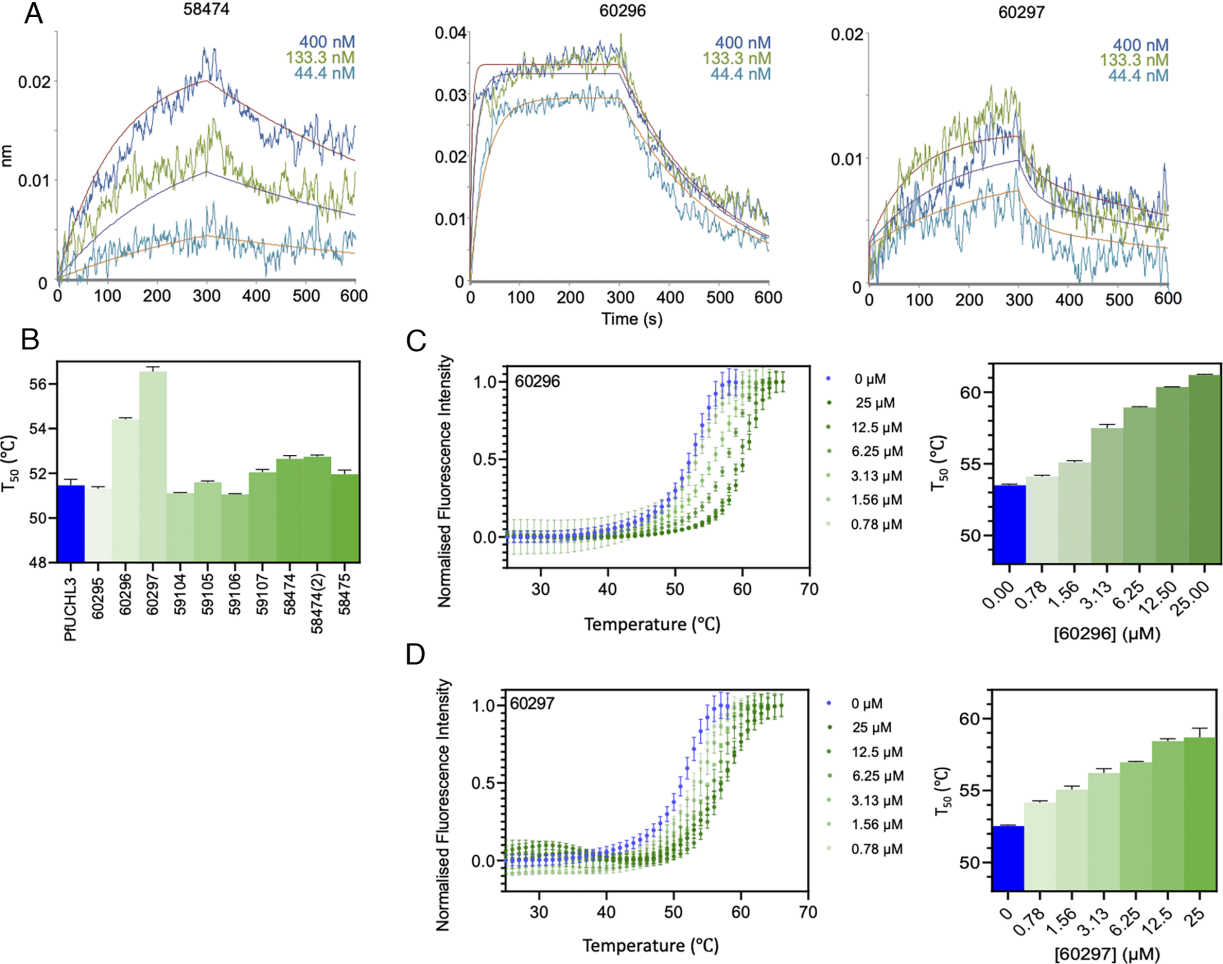
Identification of two lead peptides by ranking of their PfUCHL3-binding affinities. (*A*) BLI data: Streptavidin–biotin immobilized protein dipped into peptides at the concentrations shown for 300 s and then into buffer for 300 s. Global fits are to a 1-site model for 58474 and 60296 and a 2-site model for 60297. (*B*) Thermal shift data for 5 μM PfUCHL3 alone or incubated with each of the 10 peptides at 6 μM. (*C*) Thermal shift data for PfUCHL3 (5 μM) alone (blue) incubated with serial twofold dilutions of peptide 60296 (*C*) and peptide 60297 (*D*) from 25 μM to 0.78 μM (dark green to light green).

As an orthogonal approach, we investigated binding affinity of the 10 peptides by thermal shift assay (TSA). The melting temperature of PfUCHL3 is 51.6 °C and a range of melting temperature shifts was observed in the presence of each peptide (Fig. 2*A*). The largest stabilization was seen upon the addition of peptide 60297, which increased the melting temperature of PfUCHL3 by 5.1 °C, followed by peptide 60296 which increased the melting temperature by 3.1 °C at 25 μM. Peptides 58474 and 58474(2) were the only other two peptides to cause a significant increase in the melting temperature (1.2 °C and 1.3 °C, respectively). Peptides 59107 and 58475 only increased the melting temperature by 0.5 to 0.6 °C. Peptides 59105 and 60295 had no significant effect, and 59104 and 59106 showed small decreases in the melting temperature of −0.4 °C. Thus, overall, the trend in melting temperatures roughly follows that of the *K*_d_ values measured by BLI. To explore the effects of peptides 60296 and 60297 further, the dose dependence was measured. A concentration range of 48 pM to 25 μM was used with PfUCHL3 at a constant concentration of 5 μM, and the melting temperature was shown to increase in a dose-dependent manner (Fig. 2 *B* and *C*).

### The Peptides Are Potent and Selective Inhibitors of PfUCHL3-Mediated Ubiquitin Hydrolysis.

**Fig. 3.**
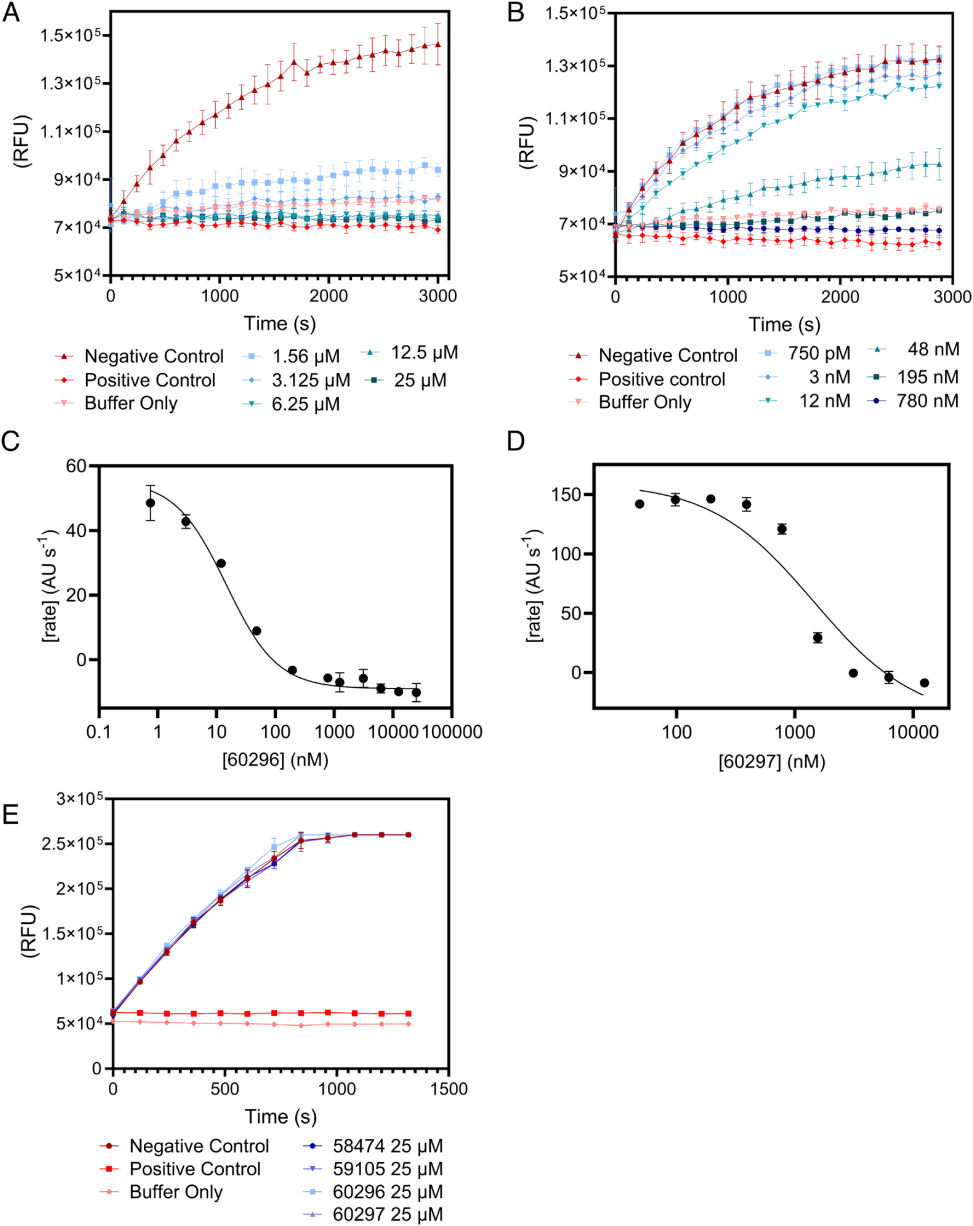
Lead peptides inhibit PfUCHL3 efficiently and specifically. PfUCHL3 (31.25 pM) activity was measured by hydrolysis of Ub-AMC substrate following incubation with serial twofold dilutions of peptide 60296 (*A*) and 60297 (*B*) “Positive Control” corresponds to N-ethylmaleimide (NEM) inhibited PfUCHL3; “Negative Control” corresponds to PfUCHL3 only (without substrate, no peptide/inhibitor); “Buffer Only” corresponds to the buffer system with no enzyme or peptides present. Maximum rate versus peptide concentration was plotted for 60296 (*C*) and 60297 (*D*). A Ub-AMC assay was performed with all peptides at an excess concentration of 25 μM incubated with HsUCHL3 (31.25 pM) to test species specificity (*E*).

Having determined that the 10 peptides bind to PfUCHL3, we next tested their ability to disrupt the enzyme’s deubiquitinating activity, as binding does not necessarily correspond to inhibition. Hydrolysis of the fluorogenic ubiquitin amido-methyl-coumarin (Ub-AMC) substrate (125 nM) by PfUCHL3 (31.25 pM) was assessed in the presence of each peptide at a concentration of 25 μM. Seven of the peptides showed no inhibition, two showed partial inhibition, and two showed complete inhibition. Ub-AMC assays were repeated for the four active peptides at different concentrations. In agreement with the BLI and TSA results, the two most potent inhibitors were peptides 60296 and 60297 which had IC50 values of 14.6 ± 2.4 nM and 1438 ± 371 nM, respectively (Fig. 3 and *SI Appendix*, Fig. S2). Importantly, all four peptides had no inhibitory effect on the human UCHL3 enzyme (Fig. 3*E*). Two control peptides were synthesized, peptides 62605 and 62606, in which the sequences were scrambled versions of peptides 60296 and 60297. Again, no inhibition was observed for these peptides even at 25 μM concentrations (*SI Appendix*, Fig. S3). Thus, the two lead peptides are sequence- and species-specific inhibitors of PfUCHL3. The selectivity for PfUCHL3 over HsUCHL3 is striking given the high sequence identity between the two proteins (*SI Appendix*, Fig. S3) particularly in the active site and the ubiquitin substrate binding site; furthermore, the sequences of ubiquitin from the two species differ by only 1 amino acid, and thus the enzyme–substrate interfaces are very similar.

### The Peptides Bind to the Ubiquitin-Interaction Interface on PfUCHL3.

**Fig. 4.**
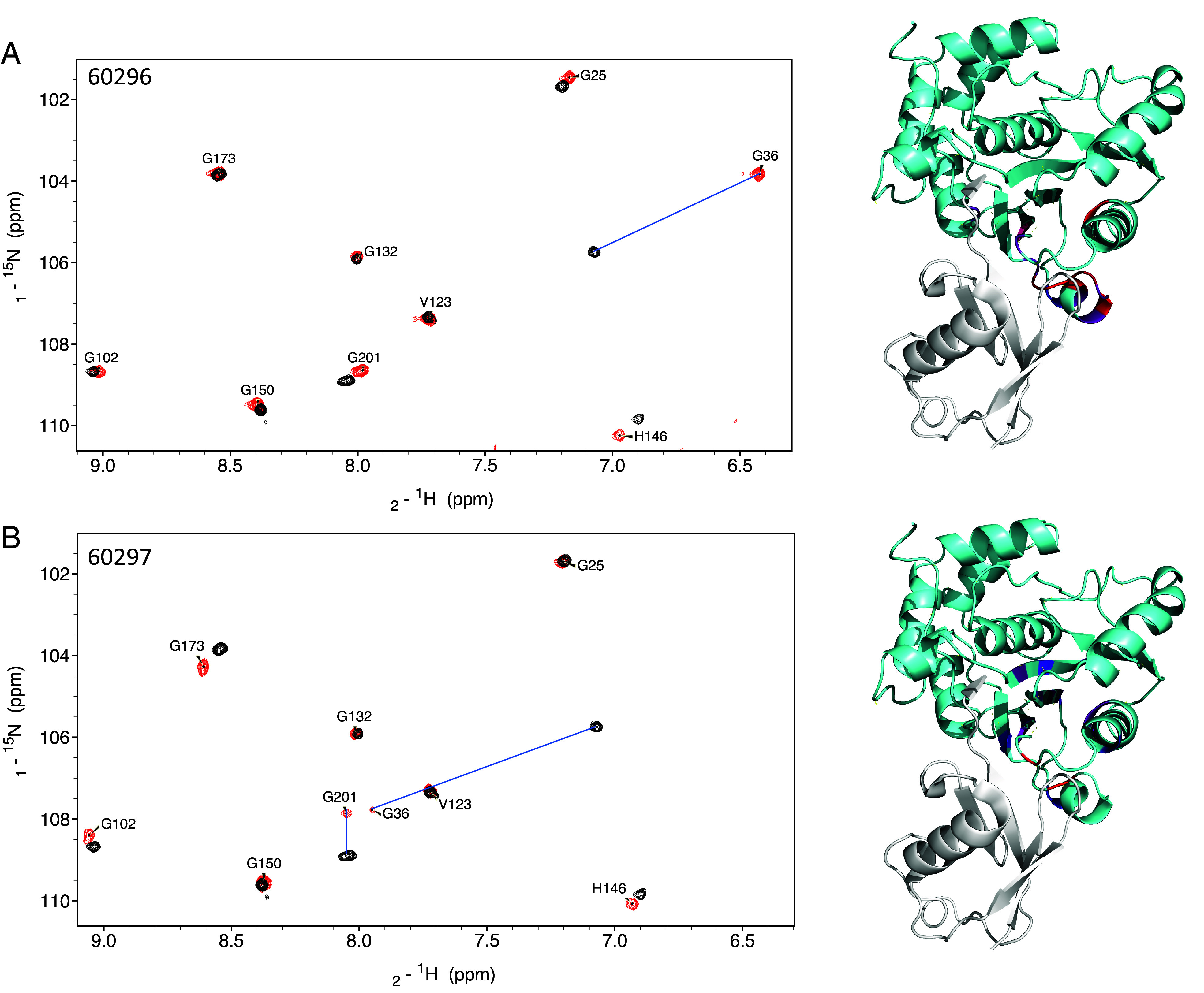
NMR spectroscopy of PfUCHL3 maps the binding site of the peptides. ^1^H–^15^N HSQC spectra of PfUCHL3 without (red) or with (blue) the addition of peptide 60296 (*A*) or peptide 60297 (*B*) (ratio 1:1). The changes in peak positions for residues that undergo changes in chemical shift of more than two SDs are indicated. Images on the *Right* show the crystal structure of PfUCHL3 (cyan) in complex with Ub (gray) [PDB code: 2WDT (12)] with the PfUCHL3 residues that show significant chemical shift changes upon peptide binding highlighted in heat map coloration. See also *SI Appendix*, Figs. S5 and S6.

To understand the mode of action underpinning the observed inhibition, we performed NMR experiments. First, we assigned the 2D ^1^H-^15^N transverse relaxation optimized spectroscopy (TROSY) spectrum acquired with ^15^N-labeled PfUCHL3. We next incubated the protein with each of the two lead peptides, 60296 and 60297, at a 1:1 molar ratio. For both peptides, a specific subset of amino acids displayed chemical shift changes with some displaying very large movements in their chemical shift values (Fig. 4). This result indicates that the peptide interaction is localized, potentially including a large number of aromatic side chains and is not eliciting a global conformational change in the protein. The PfUCHL3 residues displaying a significant chemical shift upon the addition of either peptide were mapped onto the ubiquitin-bound PfUCHL3 structure. This structure reveals two major interaction interfaces on PfUCHL3 for ubiquitin, a charged interface around the active site and a distant hydrophobic interface referred to as the ubiquitin-recognition site. The electrostatic active site interaction involves the insertion of the LRLRGG sequence at the C-terminus of ubiquitin into the active site via a tunnel created by the PfUCHL3 “crossover loop.” At the hydrophobic ubiquitin-recognition site, the sequence KTLTGK of ubiquitin forms a hairpin turn that inserts into a hydrophobic pocket on the surface of PfUCHL3. For binding of both peptides, we see that the PfUCHL3 residues that displayed the largest chemical shift changes map very closely to the hydrophobic ubiquitin-recognition site (Fig. 4).

In the case of peptide 60296, we observed extensive chemical shift changes in the ubiquitin-recognition site and adjacent β-sheet structure of PfUCHL3 upon peptide binding. It is possible that the peptide is expanding the naturally occurring substrate-binding pocket to accommodate a larger binding interface between peptide 60296 and PfUCHL3. This slightly remodels the enzyme and induces structural changes in the β-sheet core, which is located in close proximity to the ubiquitin recognition region. The finding is consistent with the TSA data showing large thermal stabilization of PfUCHL3 upon peptide binding with 60296 being the most potent inhibitor. Peptide 60297 induced similar changes in PfUCHL3, albeit to a slightly lesser extent. Thus, the peptides interact with the hydrophobic pocket and compete for binding with the KTLTGK sequence of the ubiquitin substrate, thereby inhibiting activity. Additional experiments were performed whereby peptide 60296 was incubated with PfUCHL3 at a 1:0.5 molar ratio. The results showed that the bound and unbound forms of the protein were in slow exchange, which is consistent with the slow off-rates observed in the BLI experiments.

Neither 60296 nor 60297 peptides showed any inhibitory effect on the human UCHL3 enzyme. To explore the origins of this selectivity, we examined the sequence differences between HsUCHL3 and PfUCHL3 focusing on those residues that showed the largest chemical shift changes (greater than 0.15 ppm) on peptide binding (*SI Appendix*, Figs. S4 and S5). There are several that are different in HsUCHL3, and these could result in nonbinding of the peptides: of the 18 PfUCHL3 residues that were perturbed upon binding peptide 60296, nine residues (12, 34, 37, 38, 39, 40, 43, 163, and 219 in PfUCHL3) were different in HsUCHL3; of those nine, three (Ser219, Phe37, and Asn39 in PfUCHL3) had chemical shift changes higher than 0.3 ppm. Of the 19 residues that were perturbed upon binding peptide 60297, 5 residues (50, 54, 183, 189, and 219 in PfUCHL3) were different in HsUCHL3, with only residue Ser219 showing a chemical shift change higher than 0.3 ppm. Also, six out of the 23 residues of UCHL3 were identified (by Ligplot) as interacting with ubiquitin differ between PfUCHL3 and HsUCHL3 (S12-A I34-V F37-M D157-I T163-L S219-N). Based on the chemical shift changes, the peptides may interact with nonconserved residues adjacent to the ubiquitin-binding residues such as E153 and Q205, leading to the observed selectivity.

### In Silico Modeling Reveals That Peptides Block the Interaction of Ub with PfUCHL3.

**Fig. 5.**
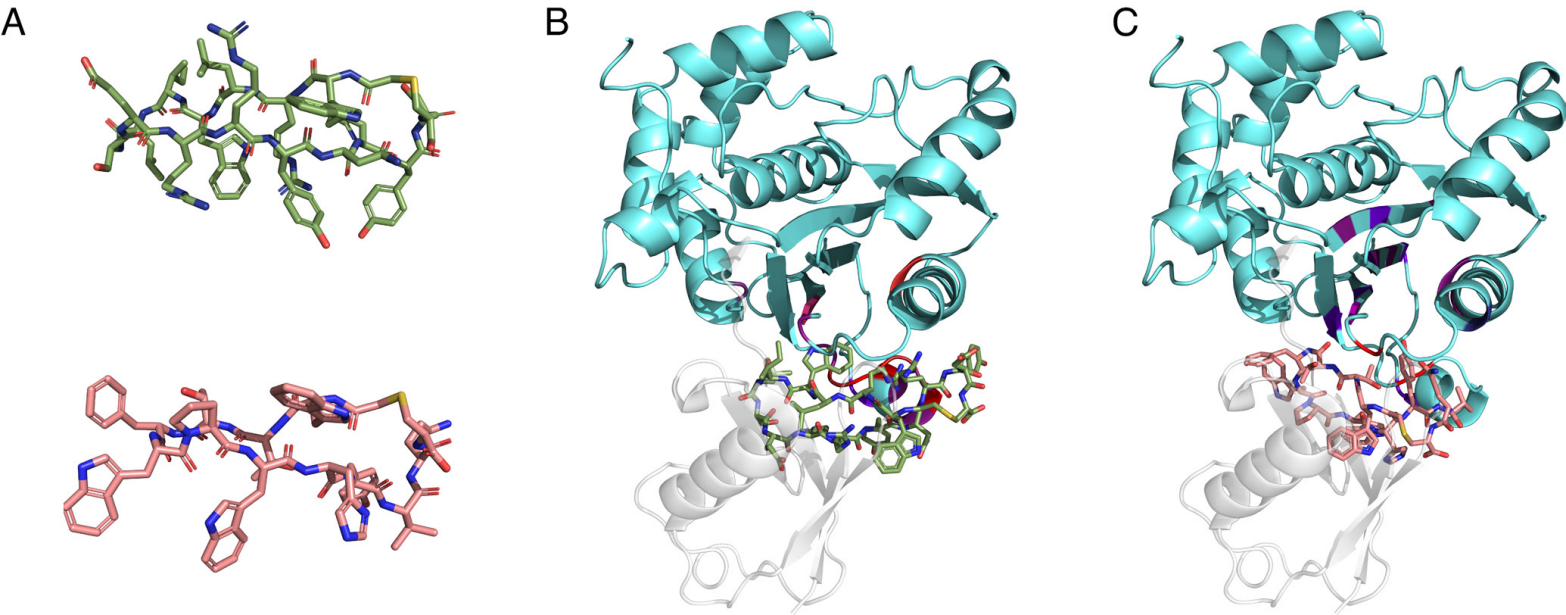
In silico modeling and docking confirm peptide binding away from PfUCHL3 active site. (*A*) The structural conformation of the cyclic peptides 60296 (*Top*) and 60297 (*Bottom*) were modeled using the Modeller v.9.25 program. PfUCHL3 (cyan) is shown in complex with the docked cyclic peptides 60296 (*B*) and 60297 (*C*) and with Ub [PDB code: 2WDT, gray (12)] to show how these peptides are interfering with the protein–protein interaction. Amino acid residues of PfUCHL3 involved in the binding as revealed by NMR are highlighted in heatmap coloring.

To provide further atomic detail on the binding sites of the peptides, we combined the NMR results with computational modeling. Peptides 60296 and 60297 were initially modeled using published cyclic peptide structures [PDB code: 6u74 (34)]. Although the crystal structures of PfUCHL3 protein are available in apo [PDB code: 2WE6 (12) and holo (Ub-bound) (PDB code: 2WDT (12)] forms, both of them lack an α-helical region (residues 59 to 76), which is 5 Å from the allosteric “ubiquitin-recognition site.” Hence, the AlphaFold (Uniprot ID: Q8IKM8) structure of PfUCHL3 protein was used to dock the peptides onto the binding site, which was defined by the surface-exposed residues of PfUCHL3 with significant 1H chemical shift changes. The top poses were similar for the two peptides (Fig. 5*A*), revealing that the peptide interaction interface overlaps with the ubiquitin substrate-binding interface, consistent with the NMR results. The cyclization of the peptides serves to lock them in a binding-competent conformation (Fig. 5 *B* and *C*). The 60296 and 60297 peptides utilize an aromatic residue, W5 and Y10, respectively, that anchors into a hot-spot pocket at the allosteric ubiquitin-recognition site of the PfUCHL3 protein. This would lead to direct steric hindrance of ubiquitin binding.

## Discussion

As in all eukaryotes, the ubiquitin pathway is critical to *Plasmodium* parasites for their survival, replication, and cellular homeostasis. Undoubtedly, many of the pathway components have yet to be discovered owing to this parasite’s unique biology and lack of sequence homology to ubiquitin enzymes in higher eukaryotes. The majority of those that have been identified are essential for parasite viability, expressed across multiple life-cycle stages, and possess only moderate identity to homologous human proteins. These points highlight the ubiquitin pathway as a promising target for the development of novel antimalarials.

In this study, we used the RaPID selection technique to identify cyclic peptide inhibitors of PfUCHL3, a dual activity DUB/deneddylating enzyme of *P. falciparum*, the deadliest malaria parasite. PfUCHL3 has been previously crystallized in both the apo and Ub-bound forms. The structures revealed two main points of contact between the enzyme and its cognate substrate: one in and around the active site and the other a hydrophobic pocket lined by Helix 7 and the loop preceding Helix 1, a signature interaction motif between UCH family proteases and Ub (12). Both human and *P. falciparum* UCHL3 structures reveal a cross-over loop that restricts access to the active site. Although it has been proposed that this loop may be flexible and able to swing aside to accommodate large, globular proteins [9], subsequent studies where the loop was cleaved or enlarged significantly increased the efficiency of di-Ubiquitin hydrolysis for both the parasite and human UCHL3 enzymes (11). These observations have led to the suggestion that the natural substrate for this enzyme is in fact small, with its main role being to replenish the free ubiquitin pool by cleaving adventitiously generated ubiquitin adducts such as thiols or amines (35). Although the wider biological function of PfUCHL3 is still unknown, transcriptomic and proteomic analyses reveal its presence across multiple stages of *P. falciparum* development suggesting it is functional—and therefore targetable— throughout the parasite’s lifecycle (36–38).

We identified two peptides that bind to PfUCHL3 with nanomolar affinity and are capable of inhibiting the deubiquitination activity of PfUCHL3 in vitro but not that of human UCHL3. NMR spectroscopy revealed that the peptides interact with the hydrophobic pocket proximal to the active site rather than the active site itself. Considering that a peptide would likely have to be linear in order to access the catalytic region under the cross-over loop, it is perhaps unsurprising that a screen based on cyclic peptides with structural rigidity would select for other locations on the enzyme’s surface.

Modeling indicated that binding of the two lead peptides sterically blocks ubiquitin from adopting the necessary orientation to access the PfUCHL3 active site, thereby inhibiting the enzyme’s DUB activity. We are now working to assess peptide inhibition in vivo. Localization of the human ortholog is predicted to be diffuse, spanning both cytoplasm and nucleus. Localization studies for the PfUCHL3 are largely consistent with this distribution, but this would still require a peptide to cross three membranes to access the enzyme: the erythrocyte, the parasitophorous vacuole, and the parasite plasma membranes. Although this may potentially be challenging, cyclic peptides have been previously and successfully used as inhibitors of *P. falciparum* proteins in vivo. In particular, STAD-2, a hydrocarbon-stapled peptide with antiplasmodial activity, was shown to access the cytoplasm of infected red blood cells (39). The exact mechanisms of how this peptide crosses the membrane have not been fully elucidated, but possible routes might include passive diffusion or uptake through endocytosis followed by endosomal escape. Indeed, there has been sustained interest in developing cell-penetrating peptides capable of escaping from endosomes, particularly in the context of viral drug delivery (21). *Plasmodium* presents a slightly more complex situation in that endosomes are generated by the double invagination of the PV and plasma membranes and result in double-membraned structures. Drawing from insights amassed as part of these studies, next-generation PfUCHL3 inhibitors can be designed to incorporate additional unnatural amino acids and cell-penetrating tags to improve permeability. Moving forward, we aim to develop these inhibitors to disrupt parasite growth and as tools to elucidate the biological role of this enzyme in *Plasmodium* parasites.

## Materials and Methods

### Protein Expression and Purification.

For library screening, PfUCHL3 with an AviTag™ (GLNDIFEAQKIEWHE) (for biotinylation by the enzyme BirA) followed by a 6xHis tag was cloned into a pET-28a expression vector. PfUCHL3-His6-Avi plasmid was transformed into C41 bacterial cells and induced with 1 mM IPTG overnight. Cells were pelleted, resuspended in lysis buffer (50 mM Tris-HCl buffer pH 8.0, 150 mM NaCl, 15 mM imidazole, 2 mM EDTA, and 1 mM TCEP) and lysed using an EmulsiFlex C5 homogenizer (Avestin) at 10 to 15,000 pounds per square inch. Lysate was loaded onto a HisTrap column (GE Healthcare) and eluted with lysis buffer supplemented with 300 mM imidazole. Fractions containing PfUCHL3 were pooled and diluted to 75 mM NaCl using MonoQ buffer (50 mM Tris-HCl buffer pH 8.0, 2 mM EDTA, and 1 mM TCEP), and the protein was further purified with ion exchange using a MonoQ 10/100 GL column (GE Healthcare) using a 0.02 to 1 M salt gradient in MonoQ buffer. Peak fractions were pooled, concentrated, and passed over a HiLoad 16/600 Superdex 75 pg column (GE Healthcare) equilibrated with storage buffer (25 mM Tris-HCl buffer pH 8.0, 200 mM NaCl, 2 mM EDTA, and 1 mM DTT). Fractions containing PfUCHL3 were pooled, concentrated, and flash-frozen.

For biotinylation of PfUCHL3, the protein BirA was first purified as follows: Expression plasmid encoding BirA was obtained from Paul Miller (University of Cambridge). The plasmid was transformed into Rosetta 2 cells and induced with 0.5 mM IPTG overnight. Cells were pelleted, resuspended in lysis buffer [PBS (phosphate buffer saline) without MgCl_2_, 1 mM DTT, 1 mM EDTA] containing a SIGMAFAST™ protease inhibitor tablet (Sigma) and DNaseI, and lysed using an EmulsiFlex C5 homogenizer at 10,000 pounds per square inch. Lysate was incubated with 3 mL of Glutathione Sepharose beads (GE Healthcare) pre-equilibrated with wash buffer (PBS, 1 mM DTT, 1 mM EDTA) in a glass chromatography tube and gently rotated (4 °C, 1 h) before unbound protein was flowed through and the beads were washed with wash buffer (250 mL). The bound protein was eluted in 16 mL elution buffer (50 mM Tris-HCl pH 8.0, 400 mM NaCl, 1 mM DTT, 50 mM Glutathione) in 4 mL fractions with a 10-min incubation time each. The eluted protein purity was purified further on a HiLoadTM 16/600 SuperdexTM 75 pg column pre-equilibrated with SEC buffer (50 mM Tris-HCl pH 8.0, 400 mM NaCl, 1 mM DTT). Fractions containing BirA were pooled, concentrated, and flash-frozen.

Purified BirA was added to the purified PfUCHL3 in the presence of biotin: Biomix A (0.5 M bicine buffer, pH 8.3), Biomix B [100 mM ATP, 100 mM Mg(OAc)_2_ and 500 μM D-biotin], concentrated PfUCHL3-AviTag (375 μM), PfUCHL3 buffer (25 mM Tris-HCl buffer pH 8.0, 200 mM NaCl, 1 mM DTT, 2 mM EDTA), and BirA (50 μM) were mixed in a ratio of 2.5:2.5:2.5:15:1. The reaction was incubated on tube rollers (R.T.P, 18 h) and loaded onto a HiLoad 16/600 Superdex 75 pg column pre-equilibrated with PfUCHL3 buffer. Protein purity was checked by 10% SDS-PAGE. Biotinylation was checked by western blot, and protein was concentrated and flash-frozen.

PfUCHL3 and HsUCHL3 proteins lacking the Avi-Tag were purified as described above, with the exception that the protein was incubated with TEV protease following HisTrap purification and repurified using a HisTrap column (to remove the cleaved tags and protease).

### Screening of PfUCHL3 Binding Macrocyclic Peptides with the RaPID System.

In vitro selections of PfUCHL3-binding macrocyclic peptides were performed using the RaPID system as previously reported (40, 41) with some minor modifications. In brief, the initial random mRNA library was transcribed, ligated to a puromycin linker, and purified by phenol-chloroform extraction and ethanol precipitation. The peptide–mRNA library was generated by translation reaction (150 µL) containing the methionine-deficient FIT system (42) and 50 µM ClAc-D-Trp-tRNA^fMet^_CAU_. Translation was performed at 37 °C for 30 min followed by a 12-min 25 °C step, addition of 30 μL of 100 mM EDTA, and a final 37 °C incubation for 30 min (this process encourages peptide-mRNA fusion and complete thioether cyclization).

After reverse transcription using MMLV RT RNase H- (Promega), the resulting peptide–mRNA–DNA fusions were incubated with PfUCLH3 immobilized on Dynabeads M-280 streptavidin (Invitrogen) for 30 min at 4 °C. The resultant complementary DNAs were eluted, analyzed by real-time PCR quantification, PCR amplified, and purified by phenol-chloroform extraction and ethanol precipitation. cDNA was transcribed into mRNA for the next round of selection. From the second round of selection onward, translation was performed at 5-µL scale, and six negative selections were performed preceding the positive selection (using 1 µL of untreated and 1 µL of biotin-bound Dynabeads M280 for each negative selection). Finally, the DNA populations resulting from the fifth, sixth, and seventh rounds were subjected to next-generation sequencing via MiSeq (Illumina).

### Chemical Synthesis of Peptides.

Macrocyclic peptides were synthesized and purified by Cambridge Peptides and Peptide Synthetics. Peptide purity and LCMS data are summarized in *SI Appendix*, Table S1.

### Ub-AMC Deubiquitination (DUB) Assay.

DUB assays were performed in triplicate in 384-well plates (Corning) using a CLARIOstar plus plate reader. PfUCHL3, HsUCHL3, or peptide were diluted in reaction buffer (50 mM Tris-HCl buffer, pH 7.5, 150 mM NaCl, 1 mg/mL BSA, 0.1% DMSO, and 1 mM TCEP) to 4× the desired final peptide/protein concentration (to keep DMSO concentration constant in all reactions). Protein and peptide (or 0.1% DMSO (mock)) were mixed 1:1 and incubated for 10 min at room temperature, with each enzyme complex at 2× the desired final concentration. Enzyme:peptide mixture (10 μL) and Ub-AMC (7-amino-4-methylcoumarin, Boston Biochem) (250 nM, 10 μL) were mixed in a 384-well plate. DUB activity was measured by monitoring the increase in AMC fluorescence. The kinetic trace at each concentration of peptide was corrected for background and then the initial rate of reaction was determined. The rate of reaction was plotted against the concentration of peptide to determine the IC50 using the following equation:

$$Y={\mathrm{Vmax}}^{*}X/\left(\mathrm{IC}50+X\right),$$

where Vmax is the reaction rate in the absence of inhibitor, X is the concentration of the peptide inhibitor, and IC50 is the peptide concentration when the rate was inhibited by the peptide by 50%. The data were fitted using Prism (Graphpad).

### Biolayer Interferometry (BLI).

BLI experiments were performed using a ForteBio Octet Red96 instrument at 25 °C. Measurements were performed in black 96-well plates with a well volume of 200 μL. Biotinylated Avi-tagged PfUCHL3 was prepared in a buffer containing 50 mM Tris-HCl buffer, 200 mM NaCl, 0.5 mM TCEP, pH 7.5, and loaded at 1 μg/mL for 60 s. Peptides were diluted to a 4 μM stock concentration in the same DMSO:water mix in which they were resuspended from their stock powder (approximately 4%). The final dilutions (to 400 nM, 133.3 nM, and 44.4 nM) were performed in the same buffer as for the PfUCHL3. Streptavidin-coated tips were used with a new tip for each measurement, because regeneration with PfUCHL3 attached to the tip was not possible. The association and dissociation times were 300 s. All data analysis was performed with the instrument software.

### Thermal Shift Assay.

Thermal shift assays were performed using a Bio-Rad CFX Connect qPCR instrument, whereby the unfolding is detected by fluorescence of the hydrophobic dye Sypro Orange that binds to the unfolded state of the protein. The experiments were performed in clear-bottom, half-volume, 96-well plates using final well volumes of 25 μL, PfUCHL3 concentration of 5 μM, peptide concentrations ranging from 47 pM to 25 μM, and Sypro Orange:protein concentration ratio of 1:400. PfUCHL3, Sypro orange, and peptide were diluted to 5x concentration in buffer, 1:2,000 concentration in buffer, and 4% DMSO, respectively, before being mixed in the well to a final 1x concentration in buffer. For the PfUCHL3-only control, the peptide was replaced with 4% DMSO and for the buffer control, the PfUCHL3 was replaced with buffer in the sample mix. The samples were incubated at 20 °C for 2 min before increasing by 0.5 °C every 30 s up to 90 °C. At each temperature, the fluorescence intensity was measured using an excitation wavelength of 471 nm and an emission wavelength of 570 nm. Data analysis was performed using the GraphPad Prism software.

### NMR Spectroscopy.

^15^N-labeled and ^13^C/^15^N-labeled PfUCHL3 proteins were produced by growing cells in K-MOPS minimal media supplemented with ^15^N-labeled ammonium chloride and ^13^C- glucose to obtain the desired isotope-labeled protein. The proteins were purified as described above. NMR spectra were recorded at the MRC Biomolecular NMR Centre at the Crick Institute (London) on a 950 MHz (Bruker Avance III HD 950) and a 700 MHz (Bruker Avance III HD 700) equipped with a triple-resonance cryoprobes on a 90% H2O, 10% D2O 100 μM sample containing 20 mM Tris-HCl buffer pH 7.5, 5 mM NaCl, 0.5 mM EDTA, 0.5 mM TCEP at 25 °C. Backbone assignments were carried out using HNCO, HN(CA)CO, HNCA, HN(CO)CA, HNCACB, and CBCA(CO)HN 3D heteronuclear NMR experiments on ^13^C/^15^N-labeled samples using standard Bruker pulse programs. Topspin (Bruker) was used for data processing and the POKY software package (43) was used for data analysis. Backbone assignments were initially made automatically using MARS (44) and completed manually. Protein:peptide samples were prepared in a 1:1 ratio unless stated otherwise. Chemical shift perturbations (CSPs) induced by peptide binding were calculated as the rmsd of the changes of the H and N chemical shifts.

### In Silico Modeling.

To build models of our cyclic peptides, we searched the PDB for structures of cyclic peptides with disulfide linkages similar to those used here. The cyclic peptide in the highest resolution (1.85 Å) complex was one in complex with acetyllysine-binding bromodomain proteins BRD4 [PDB ID: 6u74 (34)] and was used as a template to build all three cyclic peptides of interest (59105, 60296, and 60297) using Modeller v.9.25 program (45). The model with the lowest discrete optimized protein energy (DOPE) score (46) was selected. Next, a control docking experiment was performed to validate the docking protocols in Autodock Vina (47) using the BRD4–cyclic peptide complex (PDB ID: 6u74). The centroid of the grid box sized 35 Å × 35 Å × 35 Å was defined by the BRD4 residues within 4 Å of the cyclic peptide. rmsd of the atoms that make important interactions, namely O and N atoms, between the docking pose and the cyclic peptide from the crystal structure was calculated. The docking poses from Autodock Vina were then reranking using the nnscore (48) and rfscore (49) in the ODDT package (50). The scoring function that correlated to the rmsd was chosen. The docking between the cyclic peptide and BRD4 correctly modeled the binding interaction to within 0.4 Å of that in the published crystal structure.

The docking was then performed on the AlphaFold (51) model structure of PfUCHL3 protein (Uniprot ID: Q8IKM8) and the cyclic peptides. The same grid box size but with the centroid defined by residues that was marked as 1 for LW or higher than 0.15 ppm for dH in the NMR experiment was used.

## Supplementary Material

Appendix 01 (PDF)

## Data Availability

NMR data deposition data have been deposited in BMRB (BMRB ID is 52270) (52). All other data are included in the manuscript and/or *SI Appendix*.
