# Supplementary material for: Targeting the Plasmodium falciparum UCHL3 ubiquitin hydrolase using chemically constrained peptides
Source: Proc Natl Acad Sci U S A. 2024 May 13;121(21):e2322923121. doi: 10.1073/pnas.2322923121 (PMC11126973; doi:10.1073/pnas.2322923121)
Supplement: Supplementary file 1 — Appendix 01 (PDF) [file pnas.2322923121.sapp.pdf]

SUPP FIGURE 1

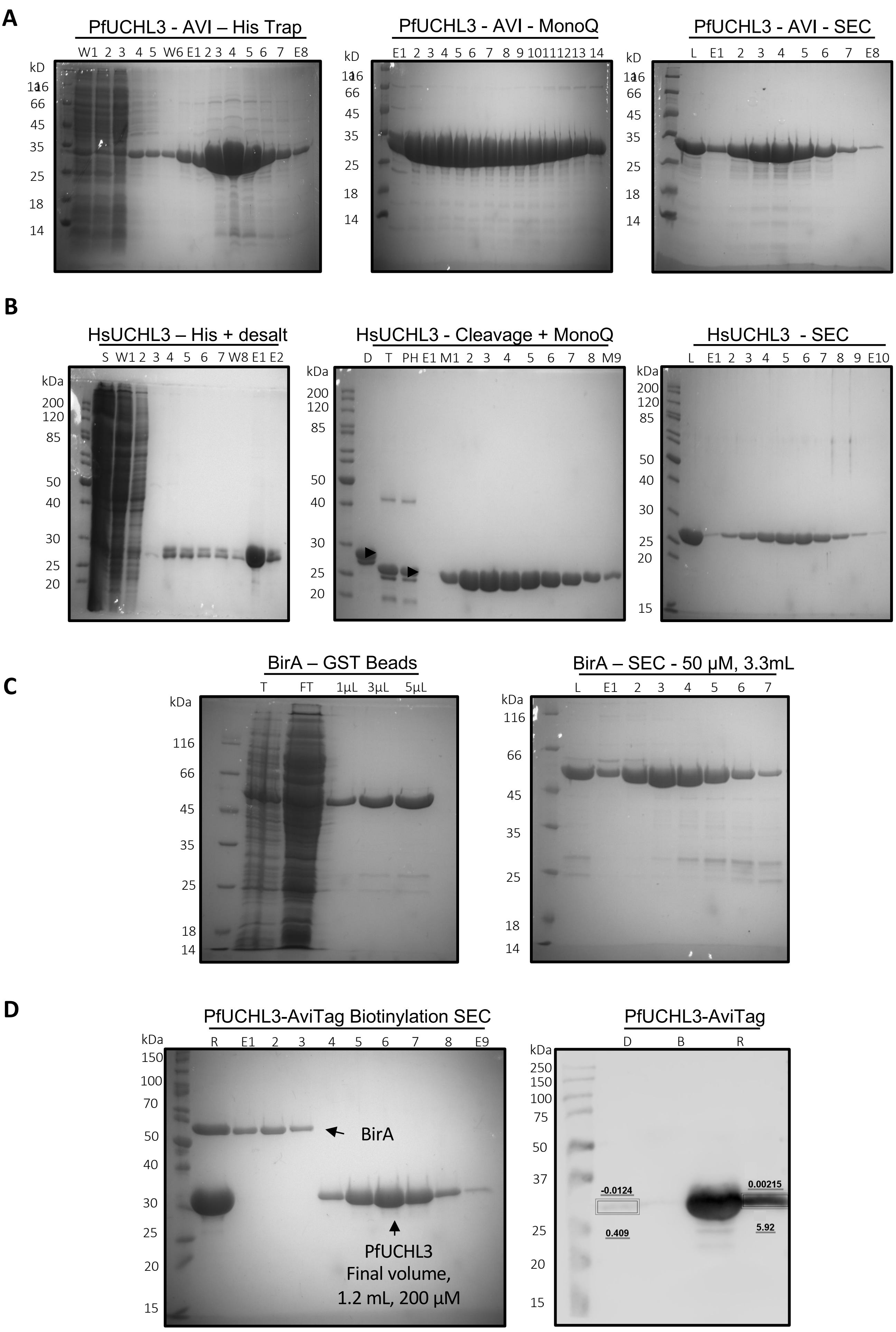

Supplementary Figure 1. ***Purification of proteins used throughout this study.*** A) SDS PAGE gels showing the purification of PfUCHL3-AviTag. Left: His-trap purification; middle: ion-exchange step; Right: size-exclusion chromatography. B) The same gels as in (A) for the purification of HsUCHL3-AviTag. C) SDS PAGE gels showing the purification of BirA-GST tag. Left: purification by GST beads; Right: size-exclusion chromatography. D) Left: SDS PAGE of the purification of the PfUCHL3 enzyme from the biotinylation mix; Right: Western blot showing the successful biotinylation of PfUCHL3 and capture of the protein on streptavadin beads.

SUPP FIGURE 2

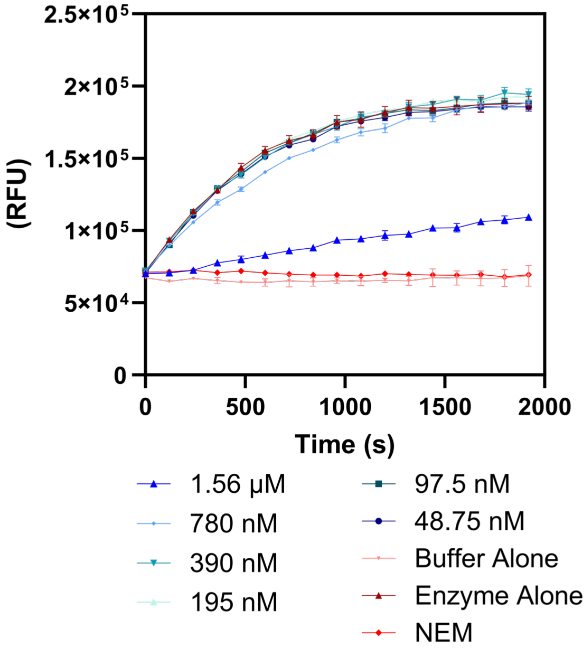

Supplementary Figure 2. ***Titration of peptide 60297 inhibition at low concentrations as measured by Ub-AMC assay.*** PfUCHL3 (31.25 pM) incubated with serial 2-fold dilutions of peptide 60297 at concentration ranging from 1.56 µM to 48.75 nM. 'Positive Control' corresponds to N-ethylmaleimide (NEM) inhibited PfUCHL3; 'Negative Control' corresponds to PfUCHL3 only (without substrate, no peptide/inhibitor)'; 'Buffer Only' corresponds to the buffer system with no enzyme or peptides present.

SUPP FIGURE 3

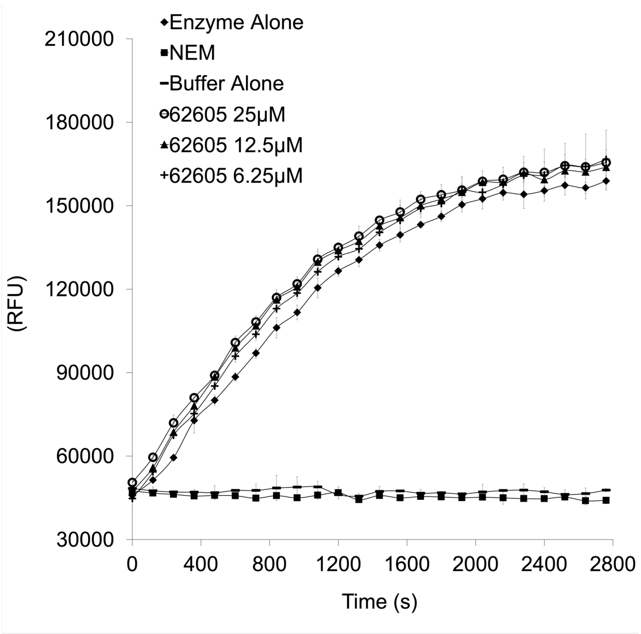

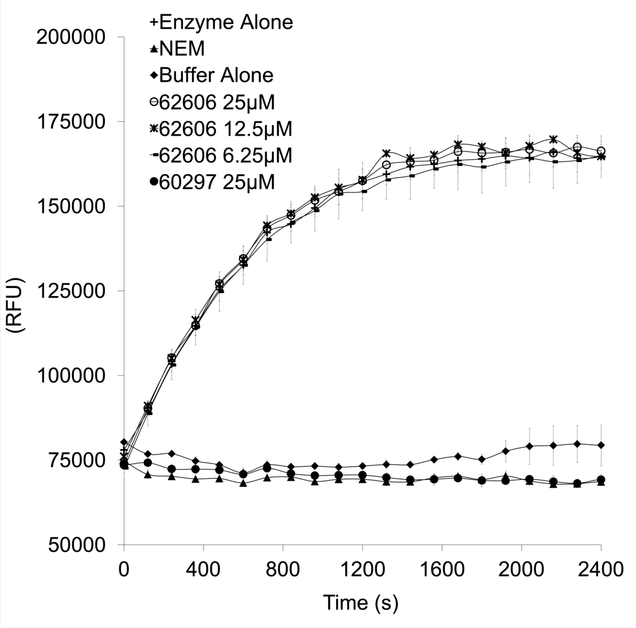

Supplementary Figure 3. *Ub-AMC assays of PfUCHL3 (31.25 pM) incubated with the scrambled peptides 62605 and 62606 (25 μM) as additional negative controls.* PfUCHL3 (31.25 pM) activity was measured by hydrolysis of Ub-AMC substrate following incubation with uM concentration of scrambled versions of the two lead peptides. NEM was included as a positive control for inhibition, whereas enzyme and buffer alone served as negative controls. Peptides 62605 and 62606 represent scrambled peptide sequences of 60296 and 60297, respectively. Their ability to inhibit PfUCHL3 activity was tested by Ub-AMC assay to confirm that the inhibition displayed by peptides 60296 and 60297 is sequence specific and not based on the biochemical properties of the peptides.

SUPP FIGURE 4

A

```
Pf   MAKNDIWTPLESNPDSLYLYSCKLGQ-SKLKFVDIYGFNNDLLDMIPQPVQAVIFLYPVN   59
Hs   -MEGQRWLPLEANPEVTNQFLKQLGLHPNWQFVDVYGMDPELLSMVPRPVCAVLLLFPIT   59

Pf   DNIVSENN---TNDKHNLKENFDNVWFIKQYIPNSCGTIALLHLYGNLRNKFELDKDSVL   116
Hs   EKYEVFRTEEEEKIKSQGQDVTSSVYFMKQTISNACGTIGLIHAIANNKDKMHFESGSTL   119

Pf   DDFFNKVNEMSAEKRGQELKNNKSIENLHHE--FCGQVENRDDILDVDTHFIVFVQIEGK   174
Hs   KKFLEESVSMSPEERARYLENYDAIRVTHETSAHEGQTEAPSIDEKVDLHFIALVHVDGH   179

Pf   IIELDGRKDHPTVHCFTNGDNFLYDTGKIIQDKFIEKCKDDLRFSALAVIPNDNFDII   232
Hs   LYELDGRKPFPINHGETSDETLLED-AIEVCKKFMERDPDELRFNAIALSAA------   230
```

B

60296

```
Pf   MAKNDIWTPLESNPDSLYLYSCKLGQ-SKLKFVDIYGFNNDLLDMIPQPVQAVIFLYPVN   59
Hs   -MEGQRWLPLEANPEVTNQFLKQLGLHPNWQFVDVYGMDPELLSMVPRPVCAVLLLFPIT   59

Pf   DNIVSENN---TNDKHNLKENFDNVWFIKQYIPNSCGTIALLHLYGNLRNKFELDKDSVL   116
Hs   EKYEVFRTEEEEKIKSQGQDVTSSVYFMKQTISNACGTIGLIHAIANNKDKMHFESGSTL   119

Pf   DDFFNKVNEMSAEKRGQELKNNKSIENLHHE--FCGQVENRDDILDVDTHFIVFVQIEGK   174
Hs   KKFLEESVSMSPEERARYLENYDAIRVTHETSAHEGQTEAPSIDEKVDLHFIALVHVDGH   179

Pf   IIELDGRKDHPTVHCFTNGDNFLYDTGKIIQDKFIEKCKDDLRFSALAVIPNDNFDII   232
Hs   LYELDGRKPFPINHGETSDETLLED-AIEVCKKFMERDPDELRFNAIALSAA------   230
```

C

60297

```
Pf   MAKNDIWTPLESNPDSLYLYSCKLGQ-SKLKFVDIYGFNNDLLDMIPQPVQAVIFLYPVN   59
Hs   -MEGQRWLPLEANPEVTNQFLKQLGLHPNWQFVDVYGMDPELLSMVPRPVCAVLLLFPIT   59

Pf   DNIVSENN---TNDKHNLKENFDNVWFIKQYIPNSCGTIALLHLYGNLRNKFELDKDSVL   116
Hs   EKYEVFRTEEEEKIKSQGQDVTSSVYFMKQTISNACGTIGLIHAIANNKDKMHFESGSTL   119

Pf   DDFFNKVNEMSAEKRGQELKNNKSIENLHHE--FCGQVENRDDILDVDTHFIVFVQIEGK   174
Hs   KKFLEESVSMSPEERARYLENYDAIRVTHETSAHEGQTEAPSIDEKVDLHFIALVHVDGH   179

Pf   IIELDGRKDHPTVHCFTNGDNFLYDTGKIIQDKFIEKCKDDLRFSALAVIPNDNFDII   232
Hs   LYELDGRKPFPINHGETSDETLLED-AIEVCKKFMERDPDELRFNAIALSAA------   230
```

Supplementary Figure 4. *Sequence alignments of PfUCHL3 and HsUCHL3*. Catalytic site residues are in red. A) Residues in the ubiquitin-binding site are highlighted in yellow. B) Residues of PfUCHL3 (and the corresponding residues for HsUCHL3) that have a chemical shift change higher than 0.15 ppm when bound to peptide 60296 are highlighted in yellow. C) Residues of PfUCHL3 (and the corresponding residues for HsUCHL3) that have a chemical shift change higher than 0.15 ppm when bound to peptide 60297 are highlighted in yellow.

SUPP FIGURE 5

A

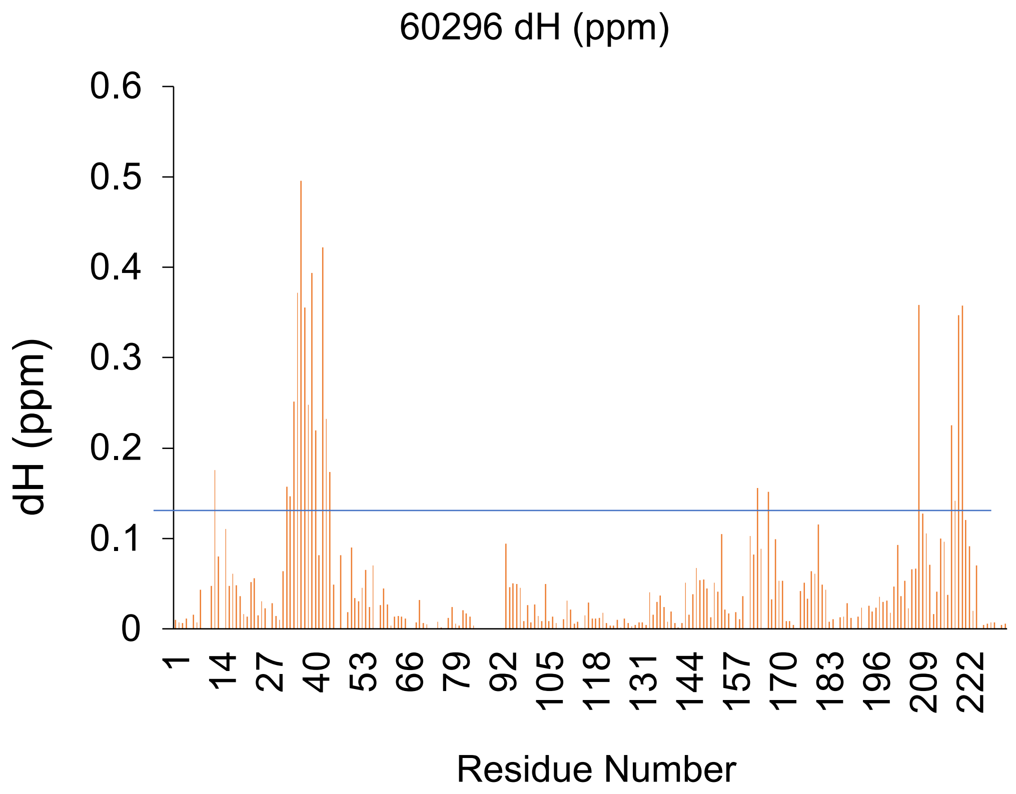

B

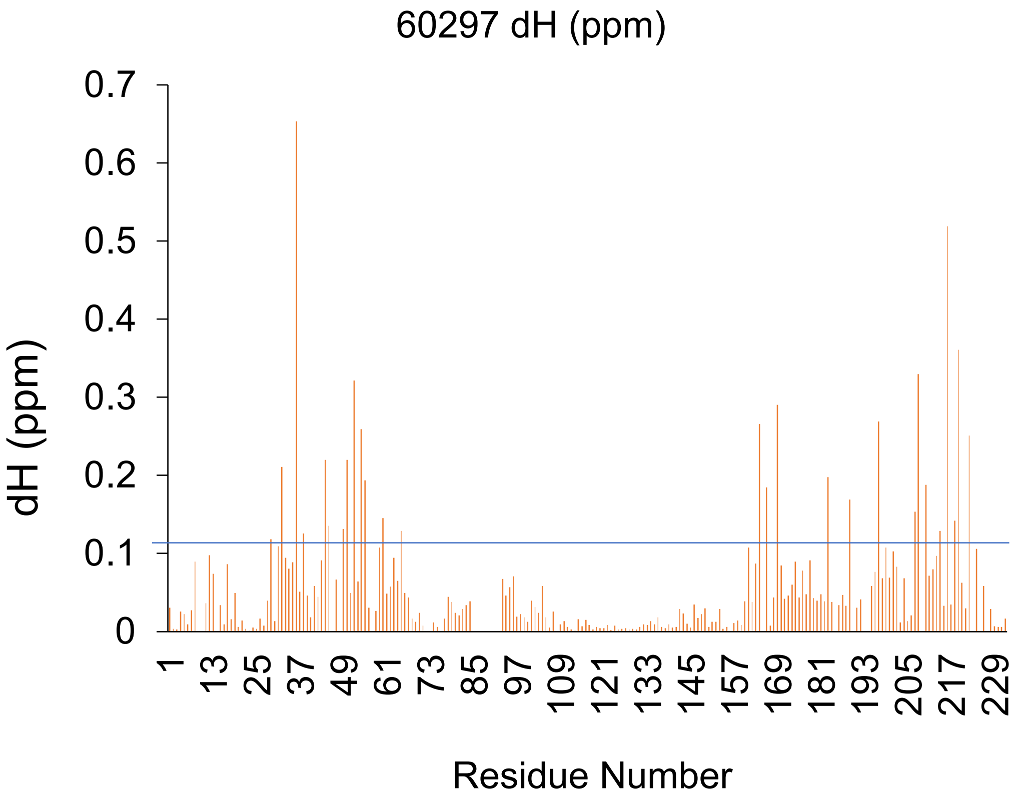

Supplementary Figure 5. *Peptide-induced chemical shifts.* Diagram showing the changes in chemical shifts of the amide protons induced in the spectrum of ^{15}N-labelled PfUCHL3 upon binding of the Peptide 60926 (left) and Peptide 60297 (right). The horizontal line indicates twice the calculated standard deviation in chemical shifts.

SUPP FIGURE 6

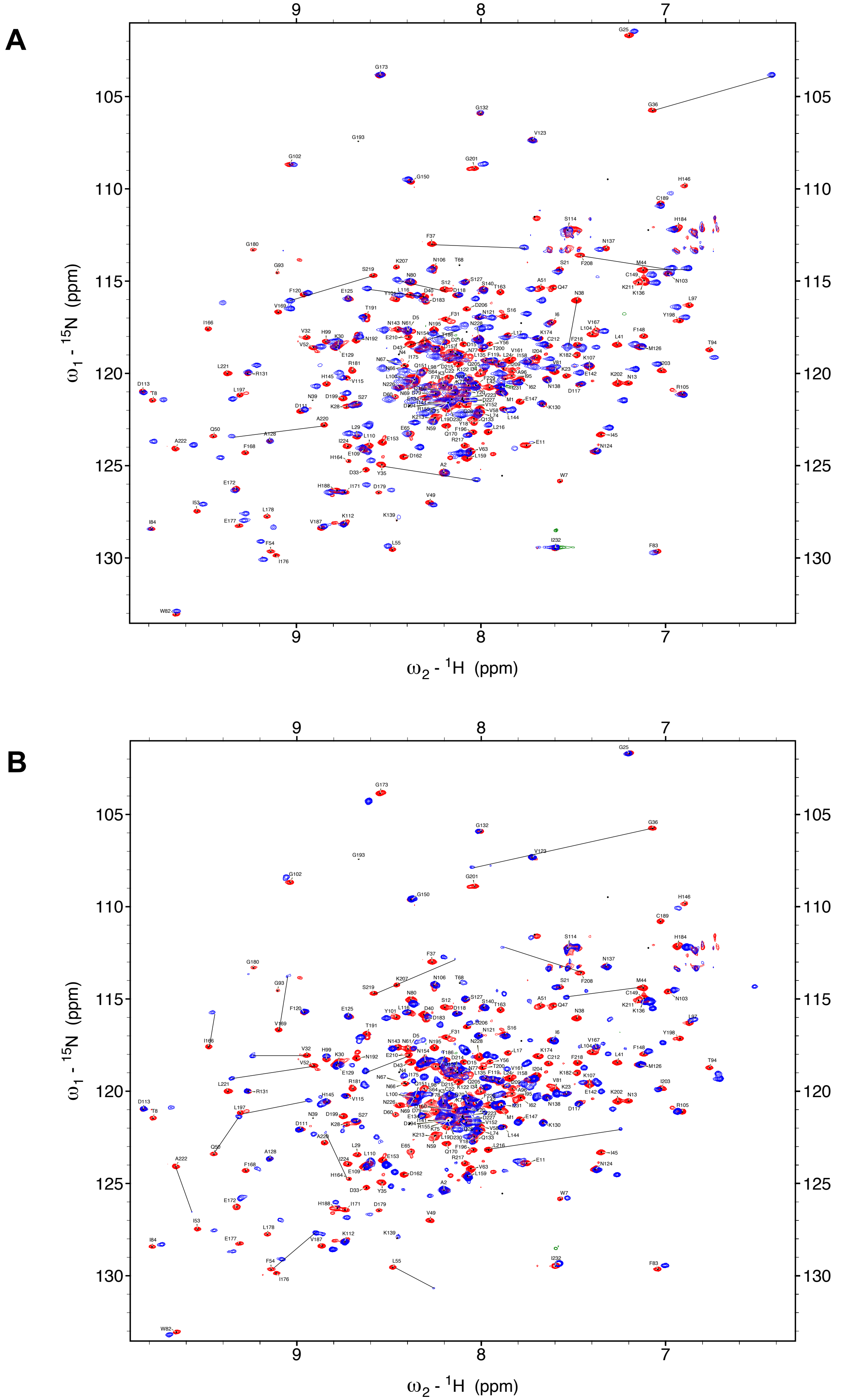

Supplementary Figure 6. NMR spectroscopy of PfUCHL3 maps the binding site of the peptides expanded view. ^{1}H-^{15}N HSQC spectra of PfUCHL3 without (red) or with (blue) the addition of peptide 60296 (A) or peptide 60297 (B) (ratio 1:1). The changes in peak positions for residues that undergo changes in chemical shift of more than two standard deviations are indicated. Images on the right show the crystal structure of PfUCHL3 (cyan) in complex with Ub (grey) (PDB code: 2WDT [12]) with the PfUCHL3 residues that show significant chemical shift changes upon peptide binding highlighted in heat map coloration.

Supplementary Table 1. Peptide purity and LCMS data for peptide synthesis.

**Peptide**	***Expected Mass (da)***	***Observed Mass (da)***	***Purity (%)***
58474	2223.5	2221.7	96.188
58474(2)	2223.5	2222.5	97.562
58475	1886.2	1884.5	99.021
59104	2362.7	2361.0	*
59105	2046.3	2046.0	95.051
59106	2124.5	2124.1	95.213
59107	1963.1	1963.4	95.402
60295	2380.7	2380.3	99.596
60296	2269.6	2269.1	95.711
60297	1693.9	1693.5	95.545
62605	2269.6	2269.1	95.973
62606	1693.9	1693.4	95.425

*peptide 59104 purity could not be determined due to solubility issues during LCMS analysis
